# Conventional and Molecular Cytogenetic Analyses in Turkish Patients with Multiple Myeloma

**DOI:** 10.5152/tjh.2011.42

**Published:** 2012-06-15

**Authors:** Beyhan Aras Durak, Olga Meltem Akay, Gülçin Sungar, Güney Bademci, Vahap Aslan, Jülide Caferler, Muhsin Özdemir, Oğuz Çilingir, Sevilhan Artan, Zafer Gülbaş

**Affiliations:** 1 Eskisehir Osmangazi University, Medical Faculty, Department of Medical Genetics, Eskisehir, Turkey; 2 Eskisehir Osmangazi University, Medical Faculty, Department of Hematology, Eskisehir, Turkey; 3 Yunus Emre State Hospital, Department of Hematology, Eskisehir, Turkey; 4 Genetiks Genetic Diagnosis Center, Istanbul, Turkey

**Keywords:** Cytogenetcis, Myeloma, FISH, Chromosomal aberrations

## Abstract

**Objective:** Multiple myeloma (MM) is characterized by the accumulation and proliferation of malignant plasma cells, secreting monoclonal immunoglobulins and genetic abnormalities in MM have implications for disease progression and survival. In the present study, we investigated the frequency of chromosomal abnormalities (CA) in Turkish patients with MM, using interphase FISH and CC and evaluated the relationship between the rearrangements detected, prognosis and stage of disease.

**Material and Methods:** We performed conventional cytogenetic and FISH studies in 50 patients to detect chromosome anomalies associated with MM. FISH probes were used to detect 13q14, 13q34, 17p13 deletions, IGH rearrangements, and monosomy and/or trisomy of chromosomes 5, 9, and 15.

**Results:** CC studies could be performed in 32 of 50 cases and five patients (15.6%) showed chromosomal aberrations while 27 (84.3%) had normal karyotypes. By FISH, eighteen percent (9/50) of cases were found to be normal for all parameters evaluated. Eighty-two percent (41/50) of the patients were positive for at least one abnormality. Chromosome 13 anomalies were detected in 54% (27/50) of cases. The second most common aberration observed is chromosome 15 aberrations (50%).

**Conclusion: **Median survival rate was shorter in patients with one of the abnormalities including chromosome 13 aberrations, IGH rearrangements or P53 deletions. Chromosome 15 aberrations were significantly higher in patients with stage III disease (p=0.02). We conclude that FISH studies should be performed in conjunction with conventional cytogenetic analysis for prognosis in multiple myeloma patients.

## Abstract (tr)

**Amaç:** Multipl Myelom (MM) plazma hücrelerinin kemik iliğinde birikmesi, proliferasyonu ve monoklonal immunglobulin sekresyonu ile karakterize klonal bir hastalıktır. Çalışmamızda Türk MM hastalarında kromozomal anomali sıklığının interfaz FISH ve klasik kromozom analizi ile belirlenmesi, saptanan yeniden düzenlenmelerle hastalığın evresi ve prognozu arasındaki ilişkinin araştırılması amaçlanmıştır.

**Gereç ve Yöntemler:** Multipl myelom tanısı alan toplam 50 olgu klasik sitogenetik ve moleküler sitogenetik (FISH) analizleri ile değerlendirilmiştir. FISH analizi ile; 13q14, 13q32, 17p13 bölgelerindeki delesyonlar, IGH bölgesini içeren yeniden düzenlenmeler ve 5, 9, 15. kromozomların monozomi ve/veya trizomileri incelenmiştir.

**Bulgular: **Konvansiyonel sitogenetik analiz olguların 32 tanesinde gerçekleştirilebilmiş olup 27 olguda (%84.3) normal karyotip izlenirken 5 olguda (%15.6) kromozom aberasyonları gözlenmiştir. FISH analizinde; olguların %18 inde (9/50) değerlendirilen tüm parametreler için normal sonuç bulunmuştur. Olguların %82 sinde ise (41/50) FISH analizi ile en az bir anomali gözlenmiştir. Olguların %54’ünde (27/50) kromozom 13 aberasyonları gözlenmiştir. İkinci en sık gözlenen aberasyon % 50 oranıyla kromozom 15 aberasyonlarıdır.

**Sonuç:** Çalışmamızda 13. kromozom aberasyonları, IGH translokasyonları ve P53 delesyonlarından en az birini bulunduran olguların ortalama median sağ kalım sürelerinin diğerlerinden daha kısa olduğu görülmüştür. Kromozom 15 aberasyonları evre 3 ile ilişkili olduğu görülmüştür (P=0.02). Çalışmamızda elde ettiğimiz verilere göre MM lı olgularda konvansiyonel sitogenetik ve özellikle FISH yönteminin hastalığın takip ve prognozunda birlikte kullanılmasının önemli olduğunu düşünmekteyiz.

## INTRODUCTION

Multiple myeloma (MM) is a common hematologic malignancy characterized by abnormal production of monoclonal immunoglobulin (IgG, IgA, IgM) or Bence Jones protein (free monoclonal k or λ light chains). Genetic changes have been implicated in the accumulation and uncontrolled proliferation of malignant plasma cells (PC) within the bone marrow. Among various prognostic factors in MM, cytogenetic abnormalities (CA) detected by conventional cytogenetics (CC) and FISH studies have emerged as a major independent parameter predicting the clinical outcome [1,2,3,4,5,6]. The most significant structural chromosomal changes are chromosome 1 structural aberrations, chromosome 13 abnormalities, translocations involving immunoglobulin heavy chain locus (IGH) of chromosome 14, trisomies and P53 gene deletions [1,2,5,6,7,8,9,10,11]. Compared to leukemias and lymphomas, cytogenetic changes are not characteristic in MM. CA in MM patients are associated with karyotypic complexity, stage and duration of disease and response to therapy [9]. The hypoproliferative nature of clonal PCs account for the great difficulty in obtaining cytogenetic data [2,3,6,9,10,11,12,13]. Abnormal karyotypes have been reported in 30-50% of patients. Karyotypes are generally complex and aneuploidy is also observed [3,5,6,9,14,15]. In the present study, we investigated the frequency of CA in Turkish patients with MM, using interphase FISH and CC. We also evaluated their relationship between clinical and laboratory features and examined their impact on overall survival.

## MATERIALS AND METHODS

50 patients who had been diagnosed with MM were enrolled. Bone marrow (BM) samples of patients from two different Hematology Centers; were referred to our Cancer Cytogenetics Section of the Medical Genetics Department from September 2005 to January 2008. The consent forms had been signed by the patients and the study has been approved by the University Ethics Committee and therefore the study was performed in accordance with ethical standards of Helsinki Declaration. 

**Sample preperation **

BM samples were cultured for 24-48 hours in RPMI- 1640 medium (supplemented with %10 fetal calf serum and antibiotics) without mitogen stimulation and with 10μg/ml colcemid concentration. Then chromosomal slides were prepared according to standard procedures (0.075 M KCl treatment and fixation with Carnoy’s fixative) [16]. Depending on metaphase availability, 20-25 metaphase cells per sample were analyzed. The clonality criteria and the karyotypic description followed ISCN (2009) recommendations [17]. 

**Slide pretreatment and denaturation **

Slides were prepared by dropping cell suspensions and then left to dry. Slides were dehydrated by the treatment of ethanol series and 0.1xSSC solution. Then the slides were treated with 2xSSC solution at 70°C and denaturated in 0.07 M NaOH solution. The slides were transferred into cold 1xSSC and then into 2xSSC solutions for 1 min. each.Then the slides were transferred into ethanol series and air-dried before hybridization [18]. 

**FISH analysis**

In the MM FISH analysis, probes for, locus specific D5S721 and D5S23 (LSI CSF1R), 9p21/CEP9 (LSI P16), D13S319 for 13q14, 13q34 (LSI13q34), CEP 15 and 17p13.1 (LSI TP53) and probes for translocation t(4;14) (p16;q32) (LSI FGFR3/IGH Dual Color, Dual Fusion Translocation Probe Set), t(11;14) (q13;q32) (LSI CCND1/ IGH Dual Color, Dual Fusion Translocation Probe Set), t(14;16)(q32;q23) (LSI IGH/ MAF Dual Color, Dual Fusion Translocation Probe Set) (Vysis, Downers Grave, IL, USA) were used. FISH was performed according to manufacturer’s specifications. 

**Microscopy**

Slides were analyzed with Olympus BX61 fluorescence microscope and images captured with a CCD camera using image analysis system (Applied Imaging). At least 200 nuclei using areas of the slides on which the cells were spread were analyzed for each probe. The cut-off points for positive values was determined for each probe from five bone-marrow samples collected from individuals with iron deficiency anemia. 

The cut-off values for each probe are as following: LSI D13S319 (13q14.3), LSI 13q34, LSI P53 (17p13.1); 8%-10%, LSI CSF1R / D5S23, D5S721 Probe Set , LSI P16 (9p21)/CEP9 Probe Set, CEP 15 ; 3%-5%, LSI FGFR3/ IGH t(4;14)(p16;q32) Dual Color, Dual Fusion Translocation Probe Set, LSI IGH/ MAF t(14;16)(q32;q23) Dual Color, Dual Fusion Translocation Probe Set, LSI CCND1/ IGH t(11;14) (q13;q32) Dual Color, Dual Fusion Translocation Probe Set; 1%. 

**Statistical analysis **

Pearson correlations test was used to identify the associations of CA detected by FISH and CC and clinical features such as age, stage, C-reactive protein, β2-microglobulin and immunoglobulin type. The overall survival (OS) curves were obtained using the Kaplan-Meier method.

## RESULTS

**Characteristics of the patients **

We evaluated 50 patients with MM, including 28 men and 22 women, median age 62 years (range, 44-78 years). Twenty-seven patients had IgG, 13 had IgA, 9 had light chain and 1 had non-secretory myeloma. At the time of enrollment, 37 patients were at Durie-Salmon stage III, 11 were at stage II and 2 were at stage I. Treatment consisted of standart-dose chemotherapy in 31 patients (melphalan/ prednisone in 9 patients, vincristine/adriamycin/dexamethasone in 21 patients and vincristine/ cyclophosphamide/ melphalan/prednisone in 1 patient) and of high-dose melphalan with autologous transplantation in 19 patients (Table 1). 

Conventional cytogenetics studies Among the 50 patients studied, cytogenetic study was done in 32 (64%). Twenty-seven patients (84%) had normal karyotypes and the remaining 5 had CA. 

**FISH studies **

Of the 50 patients, 9 (18%) had normal results, while 41 (82%) had at least one genetic aberration. FISH results are given in Table 2. 

**Chromosome 13 aberrations**

The most frequent change in our patient group involved chromosome 13. Partial or total chromosome 13 deletions were observed in 27 of 50 (54%) patients. Ten patients had monosomy 13, 8 had D13S319 and 5 had 13q34 partial deletions. Patients no.1 and no.18 had monosomy 13 plus D13S319 locus deletion, and patients no.9 and no.34 had monosomy 13 and 13q34 locus deletion. Among the 50 patients, 5 (10%) had chromosome 13 aberration as a sole abnormality, while 22 (44%) had together with other CA. 

**IGH (14q32) aberrations **

**t(4;14)(p16;q32):** 2/50 (4%) patients had t(4;14), one had a deletion of FGFR3 (4p16) and one had a trisomy of FGFR3 (4p16). 

**t(11;14)(q13;q32):** None of the patients had t(11;14) while eight had a trisomy of CCND1 (11q13). 

**t(14;16)(q32;q23):** None of the patients had t(14;16) while one had a deletion of MAF (16q23). 

We found a deletion of IGH (14q32) by three translocation probes in one patient. Other CA accompanied IGH (14q32) aberrations. 

**TP53 (17p13) deletions **

Absence of P53 at 17p13 was detected in 10/50 (20%) patients. While one patient had P53 deletion as a sole abnormality, others had this deletion with other aberrations. Chromosome 13 anomalies were the most frequent change seen together with P53 eletion.

**Numerical aberrations **

**Chromosome 5:** Numerical aberrations of chromosome 5 were detected in 15/50 (30%) patients: 13 had trisomy and 2 had monosomy. 13 patients had trisomy 5 plus hyperdiploidy. Of 2 patients with monosomy, one had isolated monosomy while one had monosomy plus a trisomy of CCND1. 

**Chromosome 9:** Numerical aberrations of chromosome 9 were detected in 13/50 (26%) patients: 9 had trisomy, 2 had tetrasomy, 1 had tetrasomy plus trisomy, 1 had monosomy. Chromosome 9 aberrations were seen as a part of hyperdiploidy except one patient with monosomy. 

**Chromosome 15:** Numerical aberrations of chromosome 15 were detected in 25/50 (50%) patients: 15 had trisomy, 8 had monosomy, 1 had trisomy plus monosomy, 1 had tetrasomy plus trisomy. Chromosome 15 aberrations were seen as a part of hyperdiploidy in 11 patients whereas 5 patients had these aberrations as a sole abnormality and 9 had together with other CA. 

**Hyperdiploidy **

Hyperdiploidy was observed in 13/50 (26%) patients by FISH, whereas it was present in only two patients by CC. Hyperdiploid karyotype was seen with chromosome 13 aberrations in 10 patients, with both chromosome 13 aberrations and P53 deletion in 2 patients and with P53 deletion in 1 patient. 

**Prognostic factors and OS**

CA in our patient group did not show any significant association with age, stage, C-reactive protein, β2-microglobulin and immunoglobulin type (p>0.05). Among the CA, chromosome 15 aberrations were found to have a significant association with advanced stage (p=0.02). Distribution of chromosome 15 aberrations according to Durie-Salmon stages is shown in Table 3.

**Figure 1:**
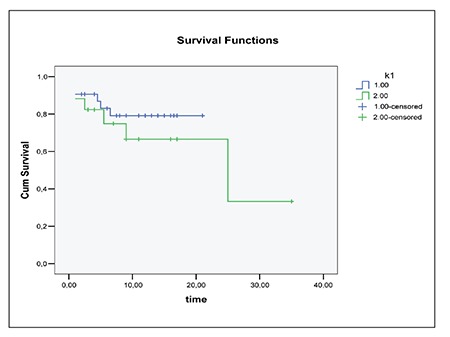
Overall survival rates of patients having at least one of the three chromosomal aberrations (chromosome 13 aberrations, IGH translocations, P53 deletion) =1 and patients having none of these aberrations=2

OS rates of patients having at least one of the three CA (chromosome 13 aberrations, IGH translocations and P53 deletion) with poor prognostic significance and patients having none of these aberrations were evaluated by Kaplan- Meier method. The median OS was shorter in patients having at least one of the three CA than the patients having none of these aberrations (Figure 1). The median OS was 17.3±1.3 months. Patients without above-mentioned CA had a median OS of 25.7±5.2 months while log rank test showed no difference between two groups according to log rank test (p<0.001) (Figure1).

## DISCUSSION

Our data showed that FISH technique has an advantage over CC in respect to its application to get better results. The success rate of chromosome banding analysis in MM patients has been reported from 29.5% to 92% in the literature [1,4,7,8,12,19,20]. In the presented study, the chromosome constitutions of 32 patients (64%) could be revealed and our success rate was within the range. Of 32 patients, 5 (15.6%) had CA. The ratio of CA in MM by CC ranged between 13.6% and 55.5% in previous studies [1,4,7,8,9,19,21,22]. The discrepancies in the genetic changes of bone marrow reflect the low in vitro mitotic activity and different infiltration degree of plasma cells. The percentage of our patients with abnormal karyotypes is similar to previous reports as given in Table 4.

Of five patients with abnormal chromosome constitutions, three had numerical sex CA whereas two had chromosome 13 and one had chromosome 1 aberrations. Depending on the karyotype analysis, not only monosomy 13/chromosome 13 deletions (Chang et al., Königsberg et al.) [7,21] but also chromosome 1 aberrations (Kaufmann et al. and Chang et al) [4,19] have been reported as the most frequently seen abnormalities. However, numerical gonosomal CA were the most frequent abnormality in the present study. This finding is consistent with the study by Cremer et al., Dewald et al. and Anwar et al. [1,8,9]. 

FISH results showed that 41 of the 50 (82%) patients had at least one abnormality, compared with a range of 36.4-86% in the literature. This high frequency of chromosomal aberrations detected in our study might be explained by the usage of a wide variety of parameters. In contrast to previous cytogenetic studies which only evaluated chromosome 13 abnormalities, we further investigated IGH rearrangements, P53 deletion, aneuploidy of chromosomes 5, 9 and 15 in addition to chromosome 13 abnormalities [1,3,4,5,6,7,8,9,12,14,15,19,21,22]. 

In FISH analysis of our patients, the most frequent abnormality was -13/13 deletion with a ratio of 54%. This percentage was higher because two different loci (13q14 and 13q34) were analyzed in this study instead of evaluation of the sole region 13q14. Therefore, we were able to determine the numerical and structural (deletions and/ or amplifications) abnormalities for two chromosome 13 specific regions could be detected. Fonseca, Anwar and Dewald have also analyzed these two loci in their series and the chromosome 13 abnormality rate was similar [1,9,14]. 

Prognostic significance of chromosome 13 abnormalities is not well known in the literature. Results of previous studies that have been concerned with this chromosome abnormality are highly diverse; chromosome 13 abnormalities were either associated with a shortened survival time or found to have an intermediate prognostic effect or had no prognostic value as a sole CA [1,11,22]. There was no significant difference between our patients having normal chromosome 13 and having chromosome 13 abnormalities as a sole genetic abnormality with respect to median survival time. Previous studies suggested that chromosome 13 abnormalities was of no prognostic value as the sole anomaly whereas it has been shown to be an important marker for poor prognosis and shorter survival time together with other CA [4,5,6,9,22]. In consistent with this finding, our patients having -13/13 deletion together with poor prognostic markers such as IGH rearrangements and P53 deletion had a shortened median OS (Figure 1). 

Numerical aberrations of chromosome 15 was observed as the second most frequent genetic change found in 50% of the patients studied by FISH analysis. The comparison of our findings with the previous studies is given in Table 3. Twenty-two of 25 patients with chromosome 15 aberrations were at Durie-Salmon stage III, that suggested an association of chromosome 15 aberrations with an advanced stage of disease. A review of the literature yielded no data relating to an association between chromosome 15 aberrations and stage. Chromosome 15 trisomies seen as a part of hyperdiploidy were found to be a good prognostic marker [5,14,23]. Due to small number of patients with an isolated chromosome 15 aberration, we could not determine the prognostic significance of chromosome 15 aberration as a sole anomaly. Further studies with larger population are necessary to determine prognostic value of chromosome 15 aberrations. 

Our patients had a lower incidence of IGH rearrangements compared to previous reports. The presented study showed that Turkish patients with MM have a different anomaly incidence if compared with the other reports (Table 4) [1,2,3,4,5,8,9,14,19,21,22]. The abnormality incidence of the presented study could not be compared since we could not find any data specific for Turkish patients. None of the patients had t(11;14) while 16% had a trisomy of CCND1 (11q13) gene region. Frequency of CCND1 gene region anomaly differs from 2.2% to 46% in the studies which may reflect a heterogeneity of the study groups or variability of the cut-off values [5,8,9,21,22]. t(4;14) and t(14;16) are known as an unfavorable prognostic factor whereas t(11;14) has a favorable prognostic value [1,3,5,9,14]. We could not determine the prognostic significance of IGH rearrangements since only two cases had t(4;14). However, patients having -13/13 deletion plus poor prognostic markers, IGH rearrangements and P53 deletion had a shortened median OS. 

Frequency of P53 deletion with a ratio of 20% in our patients was similar to previous reports. P53 deletion either as a single anomaly or accompanied by t(4;14) were linked with poor prognosis in previous studies [1,5,7,9,12,14,15,21]. P53 deletion also showed a significant association with a high level of β2- microglobulin [14]. Although P53 deletion in our patient group did not show any significant association with β2- microglobulin level, patients with -13/13 deletion having at least one of the two chromosomal aberrations (IGH translocations and P53 deletion) had a shortened median OS and poor prognosis. 

Ploidy abnormalities have been attributed to have prognostic significance in MM, which are accompanied by concurrent CA [3,9]. In patients with hyperdiploid MM, trisomies are more common while IGH translocations have a lower incidence. Hyperdiploidy appears to represent a positive prognostic factor, while patients that carry this abnormality have genetic heterogeneity. However, the presence of chromosome 13 deletions and/or 1q amplications and rarely IGH translocations influences prognosis negatively [5,9,15]. Pseudodiploidy is detected in 9-36% of MM patients whereas hypodiploidy is observed in 10-30% of patients. Partial or total monosomies; 13q, 14, 6q, 8, 16, sex chromosome deletions have been reported with respect to frequencies of occurence. Previous studies have revealed that IGH translocations and frequently chromosome 13 deletions are found in more than 85% of patients with hyperdiploid karyotype. High incidence of IGH translocations in these patients represent a negative prognostic factor [3,5,15]. Incidence of hyperdiploidy in our study group was lower than reported in two large series by Avet-Loiseau et al. and Fonseca et al. as given in Table 4. Avet-Loiseau et al. have noticed that 36% of patients with hyperdiploidy had chromosome 13 aberrations [3,12,13,14]. Hyperdiploid karyotype with chromosome 13 aberrations was seen in 92.3% of our patients, which can be explained by heterogeneity between the study cohorts and difference in the incidence of genetic changes among Turkish patients. Hyperdiploid karyotypes are associated with good prognosis in the literature [3,5,6,9,12]. 

Because all of our patients with hyperdiploidy were alive at follow-up, median OS could not be estimated. However, high survival rate of these patients might be a good prognostic indicator for MM patients. Presence of hyperdiploidy with chromosome 13 aberrations does not affect survival duration of MM patients [23]. This is further supported by our findings that hyperdiploid karyotype plus chromosome 13 aberrations showed no prognostic effect. Confirming this finding, however, requires larger studies investigating the effect of hyperdiploidy with chromosome 13 aberrations on survival rates and the role of chromosome 13 in the pathogenesis and prognosis of MM. 

The association of chromosome 13 abnormalities with age, stage, C-reactive protein, β2- microglobulin and immunoglobulin type have been compared in previous studies [7,21]. Königsberg et al reported that chromosome 13 aberrations were associated with a high level of β2- microglobulin, which suggests a poor prognosis [21]. The study of Fonseca et al. showed that patients with chromosome 13 aberrations were in advanced stage of disease [14]. We did not find any significant association between chromosome 13 aberrations and age, stage, C-reactive protein, β2- microglobulin and immunoglobulin type in our patient group. This may be due to the small number of patients in our series. In another study by Fonseca et al., the presence of chromosome 13 aberrations plus IGH translocations were found to be higher in patients without hyperdiploidy [3,7,23]. In contrast to this finding, chromosome 13 aberrations were observed in 93% of patients with hyperdiploidy in our series. 

In conclusion, we suggested that 1. CC in MM is difficult as terminally differentiated plasma cells have a low proliferation activity while FISH analysis has an advantage over CC since not only metaphases but also interphase nuclei can be analyzed and different CA can be evaluated, 2. Survival rate was shorter in patients having -13/13 deletion accompanied by poor prognostic markers, including IGH rearrangements and P53 deletions, 3. Chromosome 15 aberrations were significantly higher in advanced stages of disease which should be confirmed with further studies. 

**Conflict of Interest Statement**

None of the authors of this paper has a conflict of interest, including specific financial interests, relationships, and/or affiliations relevant to the subject matter or materials included.

## Figures and Tables

**Table 1:**
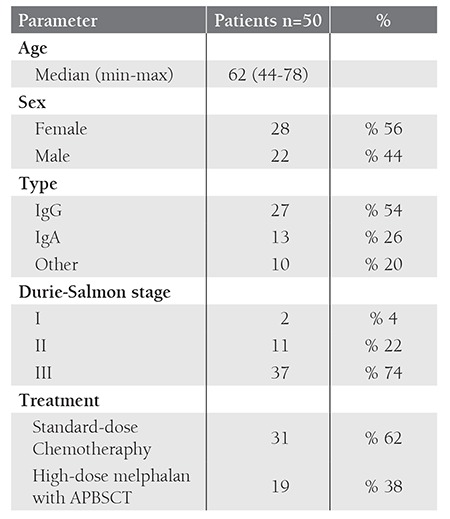
Patient Characteristics

**Table 2:**
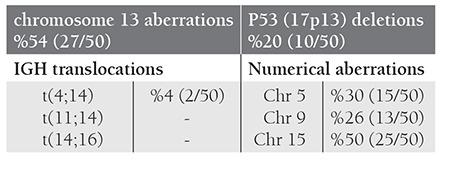
FISH Results of MM Patients

**Table 3:**
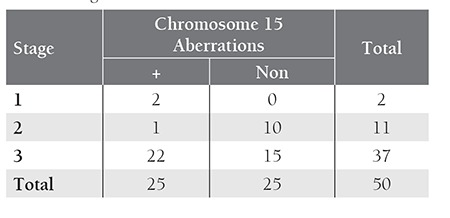
Stages of disease and chromosome 15 aberrations

**Table 4:**
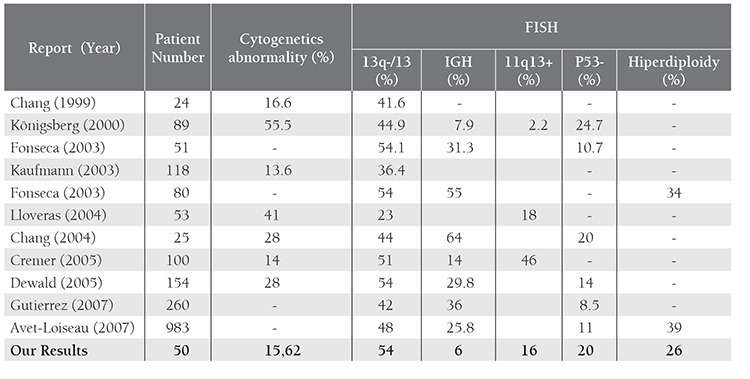
Comparison our cytogenetics and FISH results with the previos reports
